# Long-term toxicity and efficacy of FLASH radiotherapy in dogs with superficial malignant tumors

**DOI:** 10.3389/fonc.2024.1425240

**Published:** 2024-07-15

**Authors:** Bolette W. Gjaldbæk, Maja L. Arendt, Elise Konradsson, Kristine Bastholm Jensen, Sven Å. J. Bäck, Per Munck af Rosenschöld, Crister Ceberg, Kristoffer Petersson, Betina Børresen

**Affiliations:** ^1^ Department of Veterinary Clinical Sciences, University of Copenhagen, Frederiksberg, Denmark; ^2^ Department of Clinical Sciences, Lund University, Lund, Sweden; ^3^ Veterinärhuset Öresund, Limhamn, Sweden; ^4^ Department of Hematology, Oncology and Radiation Physics, Skåne University Hospital, Lund, Sweden; ^5^ Department of Oncology, Oxford Institute for Radiation Oncology, University of Oxford, Oxford, United Kingdom

**Keywords:** FLASH, radiotherapy, canine cancer, veterinary trial, ultra-high dose rate

## Abstract

**Introduction:**

FLASH radiotherapy (RT) has emerged as a promising modality, demonstrating both a normal tissue sparing effect and anticancer efficacy. We have previously reported on the safety and efficacy of single fraction FLASH RT in the treatment of oral tumors in canine cancer patients, showing tumor response but also a risk of radiation-induced severe late adverse effects (osteoradionecrosis) for doses ≥35 Gy. Accordingly, the objective in this study was to investigate if single fraction high dose FLASH RT is safe for treating non-oral tumors.

**Methods:**

Privately-owned dogs with superficial tumors or microscopic residual disease were included. Treatment was generally delivered as a single fraction of 15-35 Gy 10 MeV electron FLASH RT, although two dogs were re-irradiated at a later timepoint. Follow-up visits were conducted up to 12 months post-treatment to evaluate treatment efficiency and adverse effects.

**Results:**

Fourteen dogs with 16 tumors were included, of which nine tumors were treated for gross disease whilst seven tumors were treated post-surgery for microscopic residual disease. Four treatment sites treated with 35 Gy had ulceration post irradiation, which was graded as severe adverse effect. Only mild adverse effects were observed for the remaining treatment sites. None of the patients with microscopic disease experienced recurrence (0/7), and all patients with macroscopic disease showed either a complete (5/9) or a partial response (4/9). Five dogs were euthanized due to clinical disease progression.

**Discussion:**

Our study demonstrates that single fraction high dose FLASH RT is generally safe, with few severe adverse effects, particularly in areas less susceptible to radiation-induced damage. In addition, our study indicates that FLASH has anti-tumor efficacy in a clinical setting. No osteoradionecrosis was observed in this study, although other types of high-grade adverse effects including ulcer-formations were observed for the highest delivered dose (35 Gy). Overall, we conclude that osteoradionecrosis following single fraction, high dose FLASH does not appear to be a general problem for non-oral tumor locations. Also, as has been shown previously for oral tumors, 30 Gy appeared to be the maximum safe dose to deliver with single fraction FLASH RT.

## 1 Introduction

FLASH radiotherapy (RT) is usually characterized by radiation delivery at ultra-high dose rates greater than 40 Gy/s unlike conventional RT which delivers radiation at ~0.1 Gy/s (1). Several studies have shown a normal tissue sparing effect with FLASH RT *in vivo* (1–7) and an initial study by our group describing feasibility and early data from a heterogenous group of canine cancer patients suggested that single fraction high dose FLASH was safe in this setting (8). However, a follow-up study by our group as well as another study investigating the safety and efficacy of single fraction high dose FLASH therapy in veterinary patients (canine and feline, respectively) showed a risk of osteoradionecrosis (ORN) development when treating the oral/nasal area (9, 10). Four out of six canine patients that lived 5-6 months post irradiation experienced ORN, whilst three out of seven cats developed ORN 9-15 months post irradiation in the feline study. Both studies suggest that the high dose (and hotspot formation) could be the cause of ORN. However, when irradiating pig skin Rohrer Bley et al. also found that late skin toxicity was volume dependent, where a larger volume irradiated correlated with a higher grade of skin toxicity (10). Another conclusion from these studies was that single fraction high dose (≥35 Gy to the 100% isodose and 30 Gy to the 90% isodose, respectively) FLASH RT should be considered unsafe for tumor treatment in the oral/nasal area and, consequently, that hypofractionated FLASH RT should be investigated as a mean to avoid the risk of severe late adverse effects to the bone. Yet, it is well-known that the bones of the oral cavity, and especially the mandible, are more sensitive to developing ORN compared to other body locations (11), and that electron dose distributions are affected by the tissue heterogeneities present in some parts of the oral cavity. Hence, the ORN observed in these studies may not be a general problem.

In parallel with treating canine oral tumor patients with FLASH RT, we have treated canine patients with non-oral tumors. The objective of the current study was therefore to describe safety of single fraction high dose FLASH RT in patients with non-oral tumors. As a secondary objective, we also registered the treatment efficacy within the radiation field.

## 2 Materials and methods

### 2.1 Study design

The study was designed as a single-armed interventional trial to evaluate the feasibility and early and long-term safety for treatment of superficial macroscopic tumors or residual disease with high single dose FLASH irradiation. Evaluation of efficacy in macroscopic tumors was a secondary aim. Due to the exploratory nature of the study, no comparator arm was included.

### 2.2 Ethics

This study was approved by the Local Ethical and Administrative Committee at Department of Veterinary Clinical Sciences, University of Copenhagen, the Danish Experimental Animals Inspectorate (2020–15–0201–00429), the Swedish Board of Agriculture (5.2.18-02830/2020), and the Animal Experiments Committee in Lund, Malmö (5.8.18-14316/2021).

Furthermore, the dogs’ owners were given oral information about expected outcome and potential adverse effects. The owners signed a consent form prior to treatment.

### 2.3 Canine cancer patients

Dogs with malignant tumors or residual disease from previous surgery were examined either at the oncology clinic at the University Hospital for Companion Animals (University of Copenhagen, Denmark) or at a private veterinary hospital (Veterinärhuset Öresund, Limhamn, Sweden).

The study included patients with macroscopic non-oral superficial tumors or microscopic residual disease that was either considered inoperable or where the owners declined conventional treatment options. Due to the penetrance of the beam, tumors had to be located in the skin or subcutaneous tissue. The diagnosis was confirmed by histopathology or cytology depending on clinical relevance. Patients with co-morbidities deemed to negatively affect their fitness for anesthesia were excluded. The endpoints were defined as progressive disease that affected the quality of life based on the owners’ and veterinarians’ assessment of the patient, or if the owner requested euthanasia for other reasons.

### 2.4 FLASH treatment and dosimetry

FLASH RT was administered using a clinical Elekta Precise linear accelerator with Integrity software version 1.2 (Elekta AB, Stockholm, Sweden), which was temporarily modified for electron FLASH irradiation (12, 13). The setup, beam characteristics, and dosimetric procedures are described in detail by Konradsson et al. (8). Treatments were delivered at a nominal pulse repetition frequency of 200 Hz, with dose-per-pulse values ranging from 1.8 to 2.4 Gy, resulting in nominal mean dose rates ≥360 Gy/s.

Depending on the depth of the treatment target, tissue equivalent bolus material (Elasto-Gel EP Padding, Southwest Technologies, North Kansas City, Missouri, USA) was used to assure optimal treatment of superficial tumors or microscopic disease when relevant. Margins were decided depending on tumor pathology and whether it was scars or gross tumors. Scars generally had larger margins than gross tumors. The treatment angle was determined when the dogs were positioned on the treatment couch. The setup was monitored during the treatment by using surface scanning according to Mannerberg et al. (14).

The dogs were planned to be treated with a single high dose fraction. They were mainly treated with a local curative intent, but some dogs (tumors no 10 and 12) were treated with a palliative intent as their tumors were too substantial to be covered in depth by the 10 MeV FLASH electron beam.

A medical physicist and a board-certified veterinary oncologist decided the prescription dose (maximum dose, i.e. 100% isodose) based on knowledge about tumor type and adverse effects seen in previous treatments. The treatment dose generally increased from 15-20 Gy for the initially treated dogs to 35 Gy in the dogs treated at the end of the inclusion period.

The dogs were sedated for treatment with dexmedetomidine (2 - 4 µg/kg) and butorphanol (0.2 - 0.3 mg/kg) IV. Some dogs were supplemented with propofol (1 - 4 mg/kg). All dogs had oxygen supplied by a face mask for the duration of the sedation.

### 2.5 Follow-up, response, and adverse effects

After the treatment, the dogs had follow-up visits either at the companion animal oncology clinic at University of Copenhagen or at Veterinärhuset Öresund. Follow-up visits were planned at approximately 7 days, 1 month, 3 months, 6 months, and 12 months after treatment. When needed, additional visits were scheduled. At these visits, the dogs were examined, and macroscopic tumors were measured using a caliper. Photographs were taken of the treated area, and efficacy and adverse effects were scored. At the 6- and/or 12-months follow-up visit, the treatment area was planned for radiography or a CT scan, if bone was included in the treatment field, to assess bone integrity.

Adverse effects were assessed using the VRTOG guidelines. The original guideline, VRTOG v1, from 2001 (15) was updated in 2023 to VRTOG v2 (16). VRTOG v1 scores from 0 to 3, where 0 is no change over baseline and 3 is a severe adverse effect. The guidelines were updated in v2 to include more anatomical sites and grades with the aim of assessing adverse effects more precisely. VRTOG v2 scores from 0 to 5, again with 0 as baseline. A score of 5 is defined as toxicity resulting in death or euthanasia. The data collection for this study happened before the revision of the VRTOG guidelines, so the adverse effects in this study were originally assessed using VRTOG v1. To benefit from the VRTOG v2, the adverse effects were reevaluated for this publication based on clinical records and photographs and both grading scores are included in the results. Adverse effects occurring before the 3 months follow-up visit were considered acute effects, while those occurring from 3 months and later were considered late effects.

Treatment response was evaluated using the RECIST v1.0 for solid tumors (17). The responses were characterized as complete response (CR), partial response (PR), progressive disease (PD), or stable disease (SD). CR is defined as complete disappearance of the tumor, and PR is at least 30% reduction in the longest diameter. PD is either appearance of one or more new lesions or at least 20% increase in the longest diameter. SD is the state between PR and PD. Microscopic disease was evaluated as having either visible recurrence (R) or no recurrence (NR).

Overall survival time was defined from the day of (first) treatment to the day of euthanasia for any cause. For dogs that were still alive at the time of writing, survival time was calculated from the (first) day of treatment to the most recent owner contact. Progression-free survival time was defined as the time from (first) treatment to either tumor progression or death from tumor or unrelated causes.

The tumor volume was calculated using the formula for an ellipsoid structure:

$V=\frac{4}{3}\times \pi \times a\times b\times c$ , where a = length x 0.5, b = width x 0.5, c = depth x 0.5. Tumors with only two-dimensional measurements were estimated to be symmetrical in width and depth.

## 3 Results

Fourteen privately-owned dogs were included in this study (see **Table 1**). Two dogs had two tumors, resulting in a total of 16 treatment sites including nine gross tumors and seven post-operative scars with microscopic disease. The tumor types treated were mast cell tumor (MCT, n=7), soft tissue sarcoma (STS, n=5), squamous cell carcinoma (SCC, n=2), plasmacytoma (n=1), and histiocytic sarcoma (n=1). Seven dogs with nine tumors (tumors no 1, 2, 3, 4, 5, 6, 7, 11, 12) had their early toxicity and efficacy data (0-3 months) included in a previous publication from our group (8).

**Table 1:** Overview of patient and treatment parameters sorted primarily by dose and secondarily by field size.

	Breed, age, gender	Tumor type	Tumor location	Treatment intent	Tumor volume (cm^3^)	Dose max (Gy)	Bolus (cm)	Field size (cm)
1	French Bulldog, 7 y, MN	Soft tissue sarcoma	Subcutaneous tissue, right front limb	Definitive	Microscopic	15	1	8x4
2	Xoloitzcuintle, small, 10 y, FE	Soft tissue sarcoma	Subcutaneous tissue, left front limb	Definitive	Microscopic	16	1.5	2x6
3	Siberian Husky, 12 y FE	Plasmacytoma	Skin, left hind limb	Definitive	0.90	20	1	2 Ø
4	French Bulldog, 7 y, FE	Mast cell tumor	Skin, right front limb, distal antebrachium	Definitive	Microscopic	20	1.5	4x6
5	Siberian Husky, 12 y FE	Soft tissue sarcoma	Subcutaneous tissue, right hind limb	Definitive	6.41	25* 35	1	5 Ø
6	Pug, 8 y, ME	Mast cell tumor	Skin, right palpebra	Definitive	0.05	30	1	2 Ø
7	Pug, 8 y, ME	Mast cell tumor	Skin, right ear	Definitive	0.15	30	1	2 Ø
8	Golden Retriever, 10 y, MN	Mast cell tumor	Skin, left hind paw	Definitive	Microscopic	30	0	3 Ø
9	Jack Russel terrier, 9 y, ME	Soft tissue sarcoma	Subcutaneous tissue, right thigh	Definitive	Microscopic	30	1	5 Ø
10	Rottweiler, 5 y, FE	Soft tissue sarcoma	Caudal abdominal wall, satellites both caudolaterally and cranially	Palliative	171.99	30** 30	0	10x10 8 Ø
11	Labrador, 10 y, ME	Squamous cell carcinoma	Left nostril	Definitive	0.92	35	0	2x5
12	Bull terrier, 7 y, FN	Mast cell tumor	Subcutaneous tissue, right flank, infiltrating deeper muscle layers	Palliative	31.4	35	0	5 Ø
13	Bernese mountain dog, 7 y, FE	Mast cell tumor	Subcutaneous tissue, infiltrating between metacarpals on right front limb	Definitive	15.18	35	0.5	6 Ø
14	Standard Schnauzer, 8 y, ME	Squamous cell carcinoma	Skin, phalanx III, right front paw	Definitive	15.55	35	0	6 Ø
15	Nova scotia duck tolling retriever, 8 y, FE	Mast cell tumor	Skin, left hind limb	Definitive	Microscopic	35	1	8x4
16	Rottweiler, 6 y, FE	Histiocytic sarcoma	Skin, right olecranon	Definitive	Microscopic	35	1	10x4

* = 11 month between treatments, ** = 1 months between treatments.

All sites were treated with a single fraction, and two sites were re-irradiated. One dog with a macroscopic STS (tumor 5) was initially treated with a single fraction of 25 Gy, which led to a durable partial response. It was then retreated with 35 Gy after 11 months with the aim to shrink the tumor further. Tumor no 10 with a large rapidly growing high-grade STS received two treatments of 30 Gy with 30 days between treatments. The tumor was too large to fit inside the treatment field at the first treatment, so the second treatment was planned following a partial response to the initial treatment.

A detailed overview of the follow-up visits, along with information on medication and adjuvant treatment, is presented in **Supplementary Table 1**. Two patients (tumors no 12 and 16) were treated with adjunctive antineoplastic drugs following the irradiation. The patient with tumor no 12 started a tyrosine-kinase inhibitor (mastinib) one month after RT, and patient with tumor 16 started a lomustine-based chemotherapy protocol two weeks after RT.

### 3.1 Adverse effects

**Figure 1:**
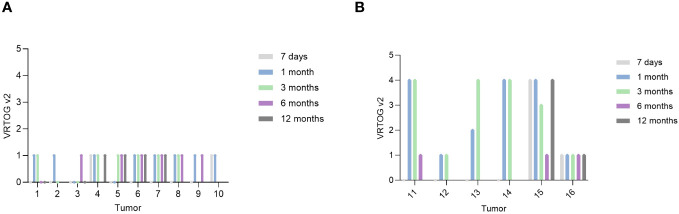
VRTOG v2 score – scoring from 0 (no adverse effects) to 5 (euthanized due to toxicity). Score 1 was assessed as mild toxicity and score 4 was assessed as severe toxicity. **(A)** Illustrates tumors that received less than 35 Gy. **(B)** Illustrates tumors that received 35 Gy. The patient with tumor 8 lost its claw capsule on digit II before the three months follow-up visit. Tumor 11 had an ulcer that did not heal until 6 months due to autotrauma (licking). Tumor 13 had an ulcer due to autotrauma (licking). The patient with tumor 14 had a small ulcer at pretreatment, furthermore the foot pad detached after one month. Tumor 15 had an ulcer present at pretreatment, which worsened after treatment.

An overview of VRTOG v2/v1 adverse effects can be seen in **Supplementary Table 2** with detailed information described in **Supplementary Table 1**. **Figure 1** illustrates the adverse effects (VRTOG v2) for each tumor at the scheduled follow-up visits.

**Figure 2:**
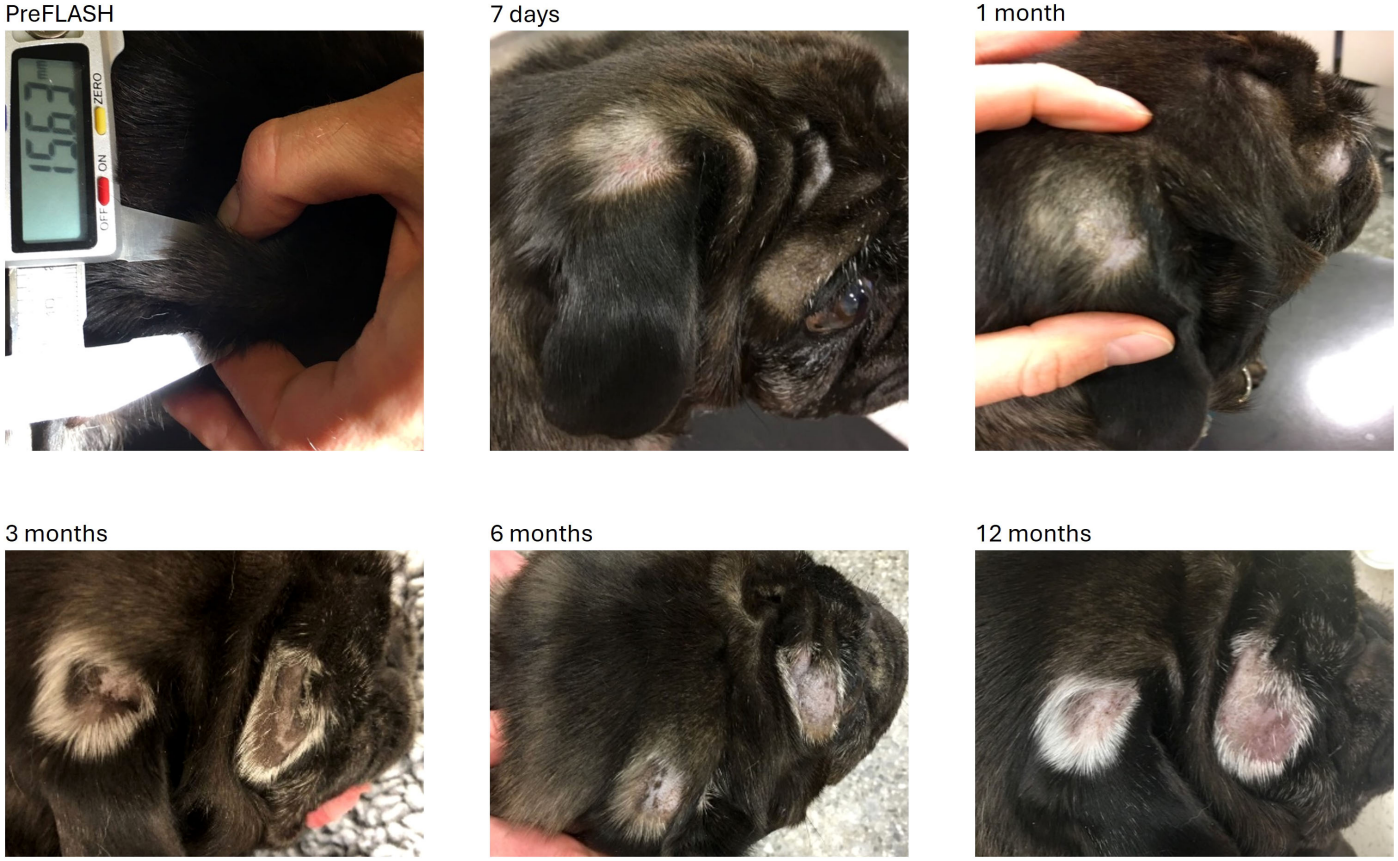
Tumors no 6 and 7 (mast cell tumors) located over the eye (6) and at the ear base (7) were both treated with 30 Gy (two fields). The alopecia at 7 days was due to clipping of the areas prior to radiotherapy. From 1 month forward the changes were graded as mild skin toxicity characterized by alopecia, leukotrichia, and hyperpigmentation.

All treatment sites treated with less than 35 Gy had no or only mild adverse effects during the follow-up period. Mild adverse effects observed included mild erythema, alopecia, dry desquamation, leukotrichia, and hyperpigmentation. **Figure 2** shows images of the dog with tumors 6 and 7, which was treated with 30 Gy to the two treatment sites and only had mild adverse effects throughout the follow-up period.

**Figure 3:**
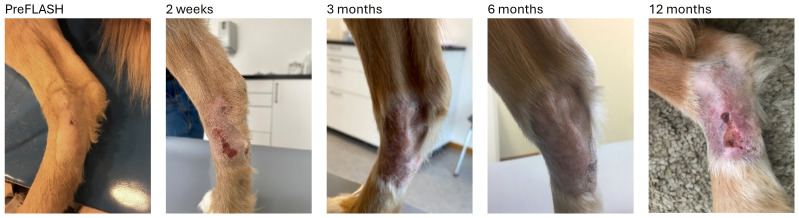
Tumor no 15, a microscopic mast cell tumor treated with 35 Gy. At preFLASH, a small ulcer was present that worsened after treatment. It healed but reappeared at 12 months. The dog was clipped for surgery and after treatment the fur did not grow back. Leukotrichia can be seen from the 3 month-timepoint.

Four out of seven sites treated with 35 Gy had a severe grade 4 (VRTOG v2) adverse effect characterized by ulcer formation. Tumors no 14 and 15 had a small ulcer present at the time of treatment that subsequently worsened. In **Figure 3**, the patient with tumor no 15 is shown with an ulceration that was present until 3 months after radiation therapy, and then reappearing at the 12 months follow-up visit.

**Figure 4:**
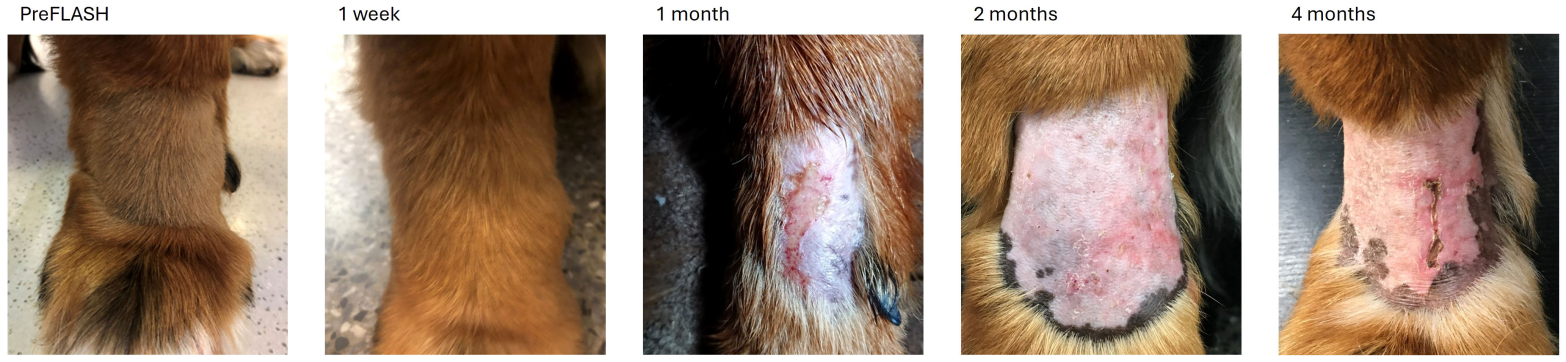
Tumor no 13, a mast cell tumor infiltrating between the metacarpal bones treated with 35 Gy. At one month after irradiation the tumor had a CR, however, due to autotrauma (licking) an ulcer had formed. When the dog was prevented from licking the ulcer, it healed but reappeared at the four-month time point due to repeated autotrama.

In two of the four treatment sites with severe adverse effects (tumors 11 and 13), the ulceration was caused or worsened by autotrauma from the dog licking the irradiated area. **Figure 4** shows dog with tumor no 13, which experienced ulceration of the skin overlying the metacarpal joint that was caused by autotrauma (licking).

When evaluating the size of the treatment field (**Table 1**), there was no clear correlation between large field size and the severity of the adverse effects (**Figure 1**). For example, tumor no 16, which was treated with 35 Gy and a 10 x 4 cm^2^ treatment field, did not develop high grade adverse effects following treatment.

Twelve dogs (13 treatment sites) had bone in the RT field, and 10 of these (11 treatment sites) were alive at 6 months (**Supplementary Table 2**). Eight of the 10 dogs (9 of 11 treatment sites) had radiographs or CT performed of the affected area (**Supplementary Table 1**). Based on imaging, there was no radiographic evidence of local ORN in these cases.

### 3.2 Efficacy and survival

**Figure 5:**
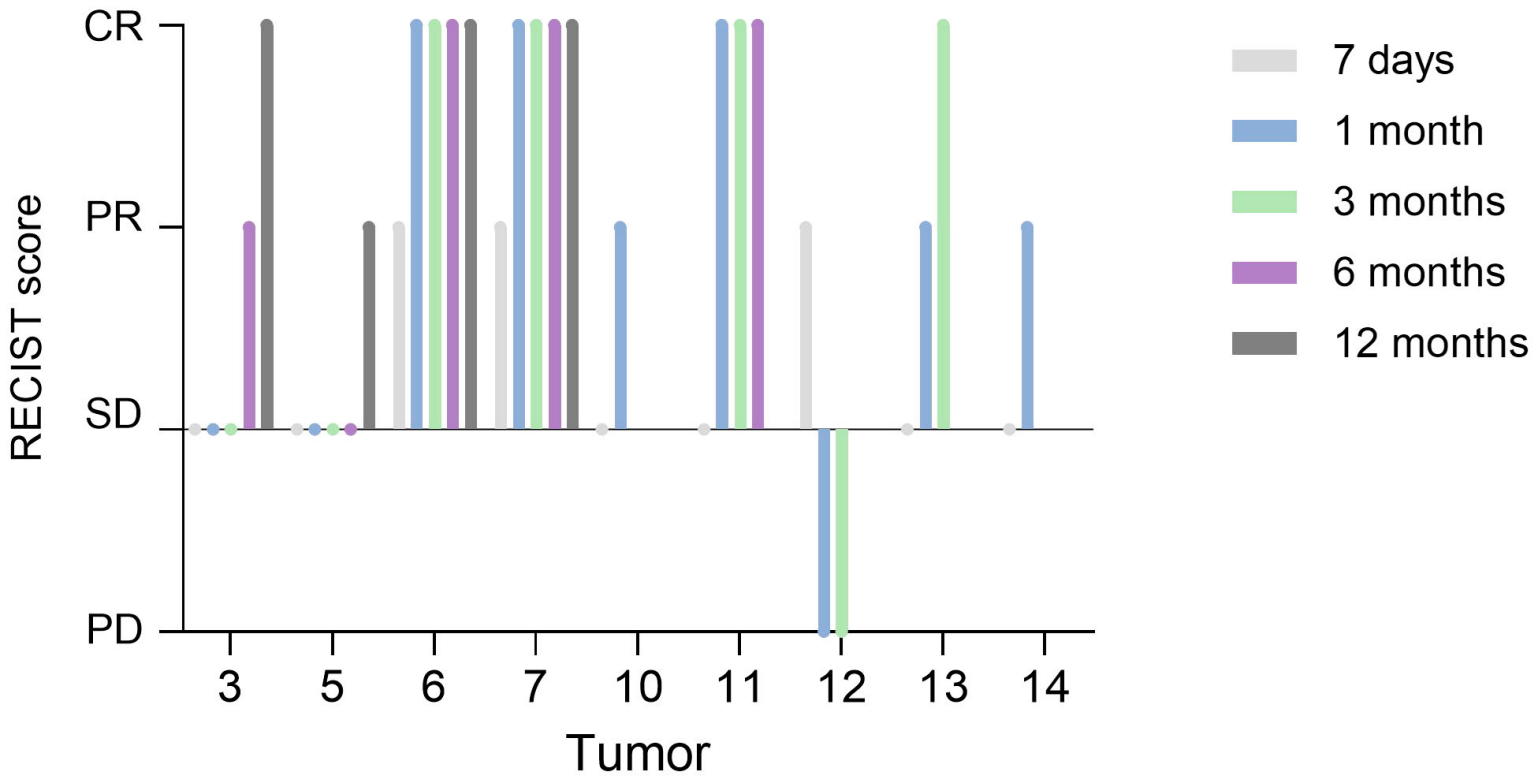
RECIST score for the macroscopic tumors. PD = progressive disease, SD = stable disease, PR = partial response, CR = complete response. Tumor 6 showed microscopic evidence of residual disease. Tumor 10 had no local progression but was euthanized due to distant metastasis (2 months post FLASH). The three patients with tumors 11, 12 and 14 had PD inside the radiation field (at 9-, 5-, and 2.9-months post FLASH, respectively) and were euthanized due to this. The patient with tumor 12 also received antineoplastic therapy (mastinib). The patient with tumor 13 was euthanized due to PD outside of the radiation field (at 4.5 months post FLASH). The patient with tumor 16 received adjunctive chemotherapy (CCNU and corticosteroids).

**Figure 5** shows treatment response for the macroscopic tumors and **Supplementary Table 3** shows treatment response, survival time and progression-free survival time for all tumors.

None of the seven treated sites with microscopic residual disease had recurrence, however, three dogs were euthanized due to unrelated disease at 21-22 months post treatment. The survival time for the dogs with microscopic disease ranged from 321 to 1196 days following irradiation.

Of the nine treatment sites involving gross disease, five had CR and four had PR. Of the five treatment sites with CR (3, 6, 7, 11, 13), one tumor (no 6, a MCT) had microscopic evidence of residual disease, due to swelling of the non-evident tumor after fine-needle aspiration. This dog was still alive at the time of writing at 1266 days (≈3.5 years) post treatment. Two other dogs with CR were euthanized due to PD (tumors no 11 inside the RT field and 13 outside the RT field), and the last dog was euthanized after 22 months due to unrelated disease (dog with tumors no 3 and 5). For the dogs with PR, three were euthanized due to PD, of which one had PD inside the RT field (tumor no 14), one had PD outside of the RT field (tumor no 12), and one had distant metastasis (tumor no 10). The dog with tumor 5 (an STS), still had a PR at the time of euthanasia for an unrelated disease at 22 months post treatment.

Five dogs were alive at the time of writing at 932 to 1266 days (≈2.5-3.5 years) post treatment (tumors no 1, 2, 6, 7, 9, 16, with tumors no 6 and 7 from the same dog). Five dogs were euthanized due to tumor progression (tumors no 10, 11, 12, 13, 14) and three dogs (tumors no 3, 4, 5, 15, with tumors no 3 and 5 from the same dog) were euthanized for tumor-unrelated causes. One dog (tumor no 8) was euthanized for unknown reasons. No dogs were euthanized due to FLASH-induced adverse effects.

## 4 Discussion

Two previous publications involving veterinary cancer patients, with tumors of the oral cavity or nasal plane, demonstrated a high risk of bone necrosis following single fraction high dose electron FLASH RT (prescribed doses of ≥35 Gy to the 100% isodose and 30 Gy to the 90% isodose, respectively) (9, 10). This emphasizes the need for caution prior to clinical implementation of FLASH RT for human cancer patients. The treatment fields for both the canine oral tumor patients (9) and the feline nasal plane SCC patients (10) included the oral cavity, which is inherently sensitive to ORN (18) and associated with tissue heterogeneities that might lead to inhomogeneous dose distributions following electron therapy. Therefore, it is important to determine if ORN following single fraction high dose FLASH RT is a general high-risk concern or pertaining mainly to treatment including the oral cavity. Accordingly, the main aim of the current study was to describe safety following single fraction high dose electron FLASH RT in a population of dogs with spontaneous non-oral tumors with efficacy as a secondary aim.

To evaluate this, we performed either planar X-ray imaging or CT imaging at least once at 6 months post-treatment or later if bone was present within the treatment field (9/11 treatment sites). Of these 11 treatment sites, four were treated with 35 Gy, while seven were treated with 30 Gy or less. There was no clinical or radiographic evidence of ORN in any of these patients. This is in contrast to our previous study, where four of six dogs with oral tumors living 6 months post-treatment developed clinical evidence of ORN (9). Similarly, the above-mentioned feline study observed ORN in 3 of 7 cats with SCC of the nasal plane after single fraction high dose FLASH RT (10). The inclusion of oral cavity bone in the treatment field was a shared feature between these two studies, as opposed to the current study. A potentially relevant difference is the common occurrence of bone invasion for oral tumors, something that was not observed in the non-oral tumors presented in the current study.

Although ORN was not observed in our patient group, other types of high-grade adverse effects were still observed in sites treated with 35 Gy. No tumor site receiving 30 Gy or less resulted in severe adverse effects. Hence, we suggest that the maximally tolerated dose for single fraction FLASH RT of non-oral tumors is 30 Gy, i.e. the same maximally tolerated dose as for oral tumors. Importantly, for the dogs that did experience high grade adverse effects, a complicating factor was the presence of ulceration at pre-treatment, walking on irradiated foot pads, or the occurrence of autotrauma, where the dog either sustained or produced a severe adverse effect through licking.

Although efficacy was not a major aim of this study, we did evaluate the treatment response, as showing safety without having anti-tumor efficacy is not clinically relevant. Overall, the treatment appeared effective for preventing recurrence in the dogs with microscopic disease. It is, however, important to note that microscopic STSs and MCTs do not necessarily recur despite no follow-up RT (19, 20) and that the follow-up time was not long enough to rule out long-term recurrence. In the nine tumors available for evaluation of response, i.e. those with macroscopic disease, 5 had a CR. The CR was durable in three smaller tumors (all <1cm^3^), while a large MCT (>15 cm^3^) and a nasal SCC progressed. However, long-term tumor control is generally not expected for canine nasal SCCs or large macroscopic MCTs treated with conventional RT alone (21–23). Four of nine tumors had a PR. Three of these were large tumors >15 cm^3^ and two of them were treated with a palliative intent, since the tumors could not be covered in depth by the treatment field. Overall, we did demonstrate some anti-tumor efficacy in this study, but despite the high single doses delivered, only a few cases had long-term tumor control. Especially larger tumors would probably benefit from an altered treatment scheme, e.g. higher total treatment doses delivered in a fractionated setting.

Oxygen was generally supplied via a face mask during anesthesia to the dogs treated in this study. The impact of oxygen on the FLASH effect is not completely clear and remains a matter of debate. However, recently published murine studies have shown the ability of oxygen supplementation to decrease or block the FLASH effect compared to room air (3, 24, 25). Meanwhile, other previously published *in vivo* studies describing a FLASH effect do not specify if the animals were supplied with 100% oxygen in relation to irradiation or not (4, 5). Perhaps, because this is considered a standard procedure for anesthetized individuals. Further research is needed to investigate if a FLASH blocking effect is also evident when irradiating larger animals such as dogs and cats. As of now, we cannot rule out that the supplementation of oxygen to the dogs treated in the current study had any effect on the FLASH effect.

There are several limitations to this study. Firstly, the treated group was small and heterogenous including various tumor types, sizes, and locations, and the dogs were treated with varying doses. Furthermore, the patients had different comorbidities resulting in euthanasia for other reasons than PD. Also, it was difficult to accurately measure tumor size, particularly in cases with complex locations and infiltration of the surrounding tissues. Importantly, the lack of a comparative conventional RT arm means that we cannot conclude if this FLASH RT protocol is more or less safe and effective compared to conventional RT for non-oral tumors. Since our aim was to conclude if single fraction high dose FLASH RT is safe (and not inducing ORN) in non-oral tumors, the information from our study setup is still valid. Yet, future studies should include conventional RT as a direct 1:1 comparison with FLASH RT, evaluating safety and efficacy in a protocol changing nothing in the two treatment arms but the dose rate.

In summary, we did observe antitumor efficacy following single fraction high dose FLASH RT for non-oral tumors, but long-term tumor control was mainly observed in smaller tumors. This suggests that larger macroscopic tumors might need a different protocol, such as higher total doses delivered with (hypo) fractionated FLASH RT. Unlike results in previously published studies, no occurrences of ORN were observed here. However, severe adverse effects, such as ulceration, were still observed, particularly in patients with lesions located on the foot pad and nasal plane and when the prescribed dose was 35 Gy. Future studies should investigate FLASH RT in a direct comparison with conventional RT to further establish if FLASH RT is safer and more effective in treating spontaneous clinical tumors. Based on our results, single fraction high dose FLASH RT is a clinically relevant treatment option for patients with non-oral tumors. Absorbed radiation doses should not exceed 30 Gy, and larger tumors should be considered for a fractionated protocol, once this has been clinically established for FLASH RT.

## Data availability statement

The original contributions presented in the study are included in the article and **Supplementary Material**. Further inquiries can be directed to the corresponding authors.

## Ethics statement

The animal studies were approved by Local Ethical and Administrative Committee at Department of Veterinary Clinical Sciences, University of Copenhagen, the Danish Experimental Animals Inspectorate (2020–15–0201–00429), the Swedish Board of Agriculture (5.2.18-02830/2020), and the Animal Experiments Committee in Lund, Malmö (5.8.18-14316/2021). The studies were conducted in accordance with the local legislation and institutional requirements. Written informed consent was obtained from the owners for the participation of their animals in this study.

## Author contributions

BG: Data curation, Formal analysis, Investigation, Visualization, Writing – original draft, Writing – review & editing. MA: Conceptualization, Data curation, Investigation, Methodology, Project administration, Resources, Writing – review & editing, Formal analysis, Supervision. EK: Conceptualization, Data curation, Formal analysis, Investigation, Methodology, Project administration, Writing – review & editing. KBJ: Investigation, Resources, Writing – review & editing. SB: Resources, Writing – review & editing. PMR: Resources, Writing – review & editing. CC: Conceptualization, Funding acquisition, Investigation, Methodology, Project administration, Resources, Writing – review & editing. KP: Conceptualization, Data curation, Funding acquisition, Writing – review & editing, Methodology, Project administration, Supervision, Visualization. BB: Conceptualization, Data curation, Funding acquisition, Investigation, Methodology, Project administration, Resources, Writing – review & editing, Formal analysis, Supervision.

## References

[1] Montay-Gruel P Petersson K Jaccard M Boivin G Germond J-F Petit B. Irradiation in a flash: Unique sparing of memory in mice after whole brain irradiation with dose rates above 100Gy/s. Radiotherapy Oncol. (2017) 124:365–9. doi: 10.1016/j.radonc.2017.05.003 28545957

[2] Montay-Gruel P Acharya MM Gonçalves Jorge P Petit B Petridis IG Fuchs P. Hypofractionated FLASH-RT as an effective treatment against glioblastoma that reduces neurocognitive side effects in mice. Clin Cancer Res. (2021) 27:775–84. doi: 10.1158/1078-0432.CCR-20-0894 PMC7854480 33060122

[3] Montay-Gruel P Acharya MM Petersson K Alikhani L Yakkala C Allen BD. Long-term neurocognitive benefits of FLASH radiotherapy driven by reduced reactive oxygen species. Proc Natl Acad Sci U S A. (2019) 116:10943–51. doi: 10.1073/pnas.1901777116 PMC6561167 31097580

[4] Vozenin M-C De Fornel P Petersson K Favaudon V Jaccard M Germond J-F. The advantage of FLASH radiotherapy confirmed in mini-pig and cat-cancer patients. Clin Cancer Res. (2019) 25:35–42. doi: 10.1158/1078-0432.CCR-17-3375 29875213

[5] Levy K Natarajan S Wang J Chow S Eggold JT Loo PE. Abdominal FLASH irradiation reduces radiation-induced gastrointestinal toxicity for the treatment of ovarian cancer in mice. Sci Rep. (2020) 10:21600. doi: 10.1038/s41598-020-78017-7 33303827 PMC7728763

[6] Bourhis J Montay-Gruel P Gonçalves Jorge P Bailat C Petit B Ollivier J. Clinical translation of FLASH radiotherapy: Why and how? Radiotherapy Oncol. (2019) 139:11–7. doi: 10.1016/j.radonc.2019.04.008 31253466

[7] Velalopoulou A Karagounis IV Cramer GM Kim MM Skoufos G Goia D. FLASH proton radiotherapy spares normal epithelial and mesenchymal tissues while preserving sarcoma response. Cancer Res. (2021) 81:4808–21. doi: 10.1158/0008-5472.CAN-21-1500 PMC8715480 34321243

[8] Konradsson E Arendt ML Bastholm Jensen K Børresen B Hansen AE Bäck S. Establishment and initial experience of clinical FLASH radiotherapy in canine cancer patients. Front Oncol. (2021) 11:658004. doi: 10.3389/fonc.2021.658004 34055624 PMC8155542

[9] Børresen B Arendt ML Konradsson E Jensen KB Bäck SÅ Rosenschöld PM. Evaluation of single-fraction high dose FLASH radiotherapy in a cohort of canine oral cancer patients. Front Oncol. (2023) 13. doi: 10.3389/fonc.2023.1256760 PMC10520273 37766866

[10] Rohrer Bley C Wolf F Gonçalves Jorge P Grilj V Petridis I Petit B. Dose- and volume-limiting late toxicity of FLASH radiotherapy in cats with squamous cell carcinoma of the nasal planum and in mini pigs. Clin Cancer Res. (2022) 28:3814–23. doi: 10.1158/1078-0432.CCR-22-0262 PMC9433962 35421221

[11] Mañón VA Shum J. Osteoradionecrosis of the cervical spine: an analysis of the literature. Oral Surgery Oral Medicine Oral Pathol Oral Radiology. (2023) 135:591–5. doi: 10.1016/j.oooo.2022.08.019 36529672

[12] Lempart M Blad B Adrian G Bäck S Knöös T Ceberg C. Modifying a clinical linear accelerator for delivery of ultra-high dose rate irradiation. Radiotherapy Oncol. (2019) 139:40–5. doi: 10.1016/j.radonc.2019.01.031 30755324

[13] Konradsson E Wahlqvist P Thoft A Blad B Bäck S Ceberg C. Beam control system and output fine-tuning for safe and precise delivery of FLASH radiotherapy at a clinical linear accelerator. Front Oncol. (2024) 14:1342488. doi: 10.3389/fonc.2024.1342488 38304871 PMC10830783

[14] Mannerberg A Konradsson E Kügele M Edvardsson A Kadhim M Ceberg C. Surface guided electron FLASH radiotherapy for canine cancer patients. Med Phys. (2023) 50:4047–54. doi: 10.1002/mp.16453 37190907

[15] Ladue T Klein MK. Toxicity criteria of the veterinary radiation therapy oncology group. Veterinary Radiol Ultrasound. (2001) 42:475–6. doi: 10.1111/j.1740-8261.2001.tb00973.x 11678573

[16] Poirier VJ Keyerleber M Gordon IK Turek MM Kent MS Bentley E. ACVR and ECVDI consensus statement: Reporting elements for toxicity criteria of the veterinary radiation therapy oncology group v2. 0. Veterinary Radiol Ultrasound. (2023) 64:789–97. doi: 10.1111/vru.13291 37582508

[17] Nguyen S Thamm D Vail D London CA. Response evaluation criteria for solid tumours in dogs (v1. 0): a Veterinary Cooperative Oncology Group (VCOG) consensus document. Veterinary Comp Oncol. (2015) 13:176–83. doi: 10.1111/vco.12032 23534501

[18] O’Dell K Sinha U. Osteoradionecrosis. Oral Maxillofac Surg Clinics North America. (2011) 23:455–64. doi: 10.1016/j.coms.2011.04.011 21798443

[19] Dobson J Scase T. Advances in the diagnosis and management of cutaneous mast cell tumours in dogs. J Small Anim Practice. (2007) 48:424–31. doi: 10.1111/j.1748-5827.2007.00366.x 17559522

[20] Dennis MM McSporran KD Bacon NJ Schulman FY Foster RA Powers BE. Prognostic factors for cutaneous and subcutaneous soft tissue sarcomas in dogs. Veterinary Pathology. (2011) 48:73–84. doi: 10.1177/0300985810388820 21139143

[21] Mason SL Pittaway C Gil BP Russak OM Westlake K Berlato D. Outcomes of adjunctive radiation therapy for the treatment of mast cell tumors in dogs and assessment of toxicity: A multicenter observational study of 300 dogs. J Veterinary Internal Med. (2021) 35:2853–64. doi: 10.1111/jvim.16264 PMC8692218 34672378

[22] Thomson M. Squamous cell carcinoma of the nasal planum in cats and dogs. Clin Tech Small Anim Pract. (2007) 22:42–5. doi: 10.1053/j.ctsap.2007.03.002 17591288

[23] Lascelles BD Parry AT Stidworthy MF Dobson JM White RA. Squamous cell carcinoma of the nasal planum in 17 dogs. Vet Rec. (2000) 147:473–6. doi: 10.1136/vr.147.17.473 11093398

[24] Iturri L Bertho A Lamirault C Brisebard E Juchaux M Gilbert C. Oxygen supplementation in anesthesia can block FLASH effect and anti-tumor immunity in conventional proton therapy. Commun Med. (2023) 3:183. doi: 10.1038/s43856-023-00411-9 38102219 PMC10724215

[25] Tavakkoli AD Clark MA Kheirollah A Sloop AM Soderholm HE Daniel NJ. Anesthetic oxygen use and sex are critical factors in the FLASH sparing effect. Adv Radiat Oncol. (2024) 9:101492. doi: 10.1016/j.adro.2024.101492 38711960 PMC11070800

