# Supplementary material for: Long-term toxicity and efficacy of FLASH radiotherapy in dogs with superficial malignant tumors
Source: Front Oncol. 2024 Jul 15;14:1425240. doi: 10.3389/fonc.2024.1425240 (PMC11284943; doi:10.3389/fonc.2024.1425240)
Supplement: Supplementary file 1 [file Table_1.docx]

**Supplementary Table 1. Detailed patient, response, and toxicity information.**

| **Patient** | | **Tumor info** | **Day 7** | **1 month** | **3 months** | **6 months** | **12 months** | **Other notes** |
| --- | --- | --- | --- | --- | --- | --- | --- | --- |
| **1** | STS low grade right front limb. Surgery 3 weeks prior to treatment. Scar tissue, incomplete margins. | | No adverse effects. | Hyperpigmentation in radiation field. Hair has not grown back from surgery. | Hyperpigmentation – seems general for this dog. Hair has grown back. | No adverse effects. X-rays of leg with no abnormal findings. | No adverse effects. No signs of tumor regrowth. | Alive at 1020 days after treatment. |
| **2** | STS low grade left front limb.  Microscopic disease, had surgery 3 weeks prior to treatment | | Mild erythema. | Small, ulcerated area, interpreted as suture reaction | No evidence of side effects or regrowth. | No visit | No visit | Alive at 932 days after treatment. |
| **3** | Plasmacytoma left hind limb. Treated concurrently with tumor 11a. Tumor size: 1.6x1.2x0.9 cm^3^  Same patient as tumor no 5. | | No adverse effect. No change in tumor size. SD | No adverse effects. Tumor size 1.6x1.2x0.9 cm^3^. SD | After 2 months: Hyperpigmentation, no change in tumor size. Same after 3 months. SD. | Mild alopecia and pigment change. Tumor 0.8x0.6 cm^2^. PR (50% reduction).  X-ray: no signs of bone lysis. | 0.4 cm palpable lesion, clinically assessed to be scar tissue. CR. No adverse effects. | Euthanized at 22 months post FLASH due to unrelated disease. |
| **4** | MCT grade II/low, right front limb. Removed with incomplete margins 3 months pre-FLASH. | | Mild erythema, alopecia (clipped during radiation, but no regrowth). No signs of tumor regrowth. | Mild erythema and alopecia. No signs of tumor regrowth. | Alopecia in RT field, mild pigment changes on footpad (in RT field). No signs of recurrence locally. De novo MCT developed distantly. | No visit | Alopecia, leukotrichia | Euthanized 22 months after treatment due to unrelated disease. No signs of local recurrence at the time of death. |

RT = radiotherapy, MCT = Mast cell tumor, STS = Soft tissue sarcoma, SCC = squamous cell carcinoma, CR = complete response, PR = partial response, SD = stable disease, PD = progressive disease, FNA = fine needle aspiration, CT = Computed Tomography, NSAID = Non-Steroidal Anti-Inflammatory Drug

| **Patient** | | **Tumor info** | **Day 7** | **1 month** | **3 months** | **6 months** | **12 months** | **Other notes** |
| --- | --- | --- | --- | --- | --- | --- | --- | --- |
| **5** | | STS right hind limb, treated concurrently with tumor 11b. Tumor size 1.7x2.0x3.6 cm^3^. Same patient as tumor no 3. | No adverse effect. No change in tumor size. | No adverse effects. Tumor size 3.2x2.2x1.4 cm^3^. SD. | At 2 months mild alopecia and pigment change. Also present at 3 months. Tumor size 1.5x3.5x2.1 cm^3^. SD. | Mild alopecia and pigment change in radiation field. Tumor size 2.9x2.3x1.4 cm^3^. SD (18.6% reduction).  X-ray: No signs of bone lysis. | ReFLASH after 10 months. Tumor 1.8x2.0x1.3 cm^3^. PR (44% reduction) | 7 days after reFLASH no new adverse effects.  1 month after reFLASH mild erythema and alopecia (grade 1).  3 months after reFLASH: tumor 1.96x1.97x0.7 cm^3^. SD. Complete alopecia. Euthanized at 22 months post FLASH due to unrelated disease. |
| **6** | MCT right palpebrae, 0.7 cm Ø. Estimated 0.2 cm depth. Same patient as tumor no. 7. | | No side effects, tumor less obvious. PR. | Alopecia and dry desquamation of skin. Tumor not palpable. CR. | Alopecia, leukotrichia and hyperpigmentation. Tumor not palpable CR. | Tumor not palpable. CR. Alopecia and leukotrichia. X-rays of skull shows no signs of bone lysis. | CT scan. No palpable tumor, however, tumor area swells considerably after FNA indicating local histamine release from microscopic disease. Alopecia in RT field and leukotrichia in the edges. CT scan no evidence of underlying bone lysis. | Treatment: Prednisolone and diphenhydramine at time of treatment for some weeks.  The owner report occasional swelling in the tumor area indicative of microscopic disease.  Control visit at 24 months incl CT scan: no signs of macroscopic recurrence or bone lysis.  Alive at 1266 days after treatment. |

RT = radiotherapy, MCT = Mast cell tumor, STS = Soft tissue sarcoma, SCC = squamous cell carcinoma, CR = complete response, PR = partial response, SD = stable disease, PD = progressive disease, FNA = fine needle aspiration, CT = Computed Tomography, NSAID = Non-Steroidal Anti-Inflammatory Drug

| **Patient** | **Tumor info** | **Day 7** | **1 month** | **3 months** | **6 months** | **12 months** | **Other notes** |
| --- | --- | --- | --- | --- | --- | --- | --- |
| **7** | MCT at the base of right ear, 1.2 cm Ø. Estimated 0.2 cm depth.  Same patient as tumor no 6. | No side effects reported, tumor less obvious | Alopecia and dry desquamation in skin. Tumor not palpable. | Alopecia, leukotrichia and hyperpigmentation. Tumor not palpable. CR. | Tumor not palpable. CR. No cells on FNA. Alopecia and leukotrichia. X-rays of skull shows no signs of bone lysis. | Alopecia in RT field with leukotrichia in the edges. No palpable tumor. CR. CT no evidence of bone lysis. |  |
| **8** | MCT grade II/low, left hind paw. Removed with incomplete margins 4 weeks pre-FLASH. | No adverse effects. No signs of tumor regrowth. | Marked erythema in RT field and around nail bed. The foot pad in RT field appears less pigmented, thinner, and erythematous. No signs of tumor regrowth. | Alopecia in RT field, claw capsule on digit II has detached. The foot pad looks normal. No signs of tumor regrowth. | 1 cm Ø lichenification of the skin No signs of tumor regrowth. X-ray: no signs of bone lysis. | - | Euthanized 11 months post treatment for unknown reasons. |
| **9** | STS low grade right thigh. Incomplete deep and lateral tumor margin. Treated in microscopic setting 1.5 months post-surgery. | No side effects. No signs of tumor regrowth. | Light alopecia in RT field. | 2 months control visit: No adverse effect. No signs of tumor regrowth. | Alopecia in RT field, RT field appears tender on palpation. Scar appears irregular, no obvious masses as signs of regrowth. X-ray: no signs of bone lysis. | No visit | Alive at 1196 days after treatment. |
| **10** | High grade sarcoma caudal abdominal wall. Tumor 7.7x7.9x5.4 cm^3^. Satellites present both cranially and caudolaterally. | Tumor 6.0x5.0 cm^2^. SD (24% reduction) Mild alopecia. The satellites present at treatment are no longer palpable. | Second FLASH treatment. Tumor 4.7x5.0 cm^2^. PR from first treatment (36.7% reduction). Satellites no longer present. Alopecia, hyperpigmentation. | - | - | - | Euthanized 2 months after first treatment due to intraabdominal metastasis and coagulopathy. Tumor size on ultrasound 3.24x1.98 cm^2^. PR (59.0% reduction). |

RT = radiotherapy, MCT = Mast cell tumor, STS = Soft tissue sarcoma, SCC = squamous cell carcinoma, CR = complete response, PR = partial response, SD = stable disease, PD = progressive disease, FNA = fine needle aspiration, CT = Computed Tomography, NSAID = Non-Steroidal Anti-Inflammatory Drug

| **Patient** | **Tumor info** | **Day 7** | **1 month** | **3 months** | **6 months** | **12 months** | **Other notes** |
| --- | --- | --- | --- | --- | --- | --- | --- |
| **11** | SCC left nostril.  Tumor 2.0x1.35x0.65 cm^3^, visible from outside. Sneezing every day, occasional epistaxis. CT thorax no signs of metastasis. | No adverse effects. Reduced epistaxis, increased sneezing. | Tumor no longer visible from outside. CR. No recurrence of epistaxis, lymph node ipsilateral has become smaller, new aspirates no longer show evidence of metastasis. Still sneezing. Desquamation of nasal planum with areas of ulceration. | No visible tumor, CR. Complete depigmentation of nasal plane and alopecia in RT field and 0.5 cm Ø superficial ulcer | At 5 months CT: CR. Owner reports respiratory sound and occasional sneezing. CT shows accumulated mucous/secretions. | - | Treatment: NSAID, pregabalin, antibiotics.  Revisit after 9 months due to ulceration on nose, biopsy from lesion shows recurrence of SCC. Euthanized after 10 months due to progressive disease. |
| **12** | Subcutaneous MCT right flank. Mitotic count 5 pr. 10 HPF.  Tumor 5.0x4.0x3.0 cm^3^. Macroscopic regrowth 2 months after surgical removal with incomplete margins. | Tumor 2.7x2.5x2.0 cm^3^. No side effects. PR (46% reduction). | Alopecia, erythema, desquamation, inguinal lymph node enlarged, new mass in dorsal part of radiation field. The new mass: 3.42x2.8 cm^2^. PD. Started on masitinib. | Alopecia.  3.5 cm firm mass in caudal part of radiation field, overlying part of the surgical scar. | - | - | Treatment: Masitinib started at 1-month visit. Other treatment were diphenhydramine, prednisolone, and omeprazole.  Euthanized before 6 months control due to progressive disease. |

RT = radiotherapy, MCT = Mast cell tumor, STS = Soft tissue sarcoma, SCC = squamous cell carcinoma, CR = complete response, PR = partial response, SD = stable disease, PD = progressive disease, FNA = fine needle aspiration, CT = Computed Tomography, NSAID = Non-Steroidal Anti-Inflammatory Drug

| **Patient** | **Tumor info** | **Day 7** | **1 month** | **3 months** | **6 months** | **12 months** | **Other notes** |
| --- | --- | --- | --- | --- | --- | --- | --- |
| **13** | MCT. Dorsally to and infiltrating between metacarpals on right front limb.  Difficult to discern due to infiltration, but approx. 5x5.8x1.0 cm^3^. | Tumor SD. No adverse effects | 1 month: Marked self-trauma (licking) leading to moist desquamation. Resolves when prevented from licking. Unable to assess tumor response. 1.5 months: complete alopecia in entire RT field. Scarring of skin after previous auto-trauma. RT field oedematous and thickened. Tumor decreased in size. PR. | No palpable tumor. CR. Dog has licked the treated area again, so new small ulceration. Still complete alopecia in RT field. | - | - | Euthanized after 4.5 months due to tumor progression outside of RT field.  Other treatments: Prednisolone for two weeks after treatment, antihistamine, and ointment with betamethasone and fucidic acid. |
| **14** | SCC right front paw. Tumor 3.0x4.5x2.2 cm^3^  Small ulcer at time of treatment. Metastasis to right prescapular lymph node. | Tumor 3.5x2.5x1.2 cm^3^. SD (22.2% reduction) ulcer has not healed. | Tumor 3.0x2.0x1.0 cm^3^. PR (33.3% reduction). Tumor is difficult to discern. Foot pad (in RT field) starting to detach. | Tumor progression leading to ulceration at the site of the foot pad. Euthanized. | - | - | Treated with NSAID, antibiotics, and ointment with betamethasone and fucidic acid.  Euthanasia at 2.9 months post RT due to tumor progression. |

RT = radiotherapy, MCT = Mast cell tumor, STS = Soft tissue sarcoma, SCC = squamous cell carcinoma, CR = complete response, PR = partial response, SD = stable disease, PD = progressive disease, FNA = fine needle aspiration, CT = Computed Tomography, NSAID = Non-Steroidal Anti-Inflammatory Drug

| **Patient** | **Tumor info** | **Day 7** | **1 month** | **3 months** | **6 months** | **12 months** | **Other notes** |
| --- | --- | --- | --- | --- | --- | --- | --- |
| **15** | MCT grade II/low left hind limb.  Removed with incomplete margins 1.5 months prior to FLASH treatment.  Ulceration presents in the scar before treatment start. | Ulceration from pretreatment has worsened, erythema, dry desquamation seen in radiation field. | 2x2cm alopecic area caudal to the hock, which becomes erythematous upon palpation. | Persistent erythema. A slightly smaller superficial ulcer remains approx. 2-3 mm Ø in the proximal edge of the radiation area. | Ulcer completely healed. A small amount of leukotrichia around the radiation field and on the back of the leg.  At 8 months X-ray: no signs of bone lysis. | The skin in the RT field shows erythema. Suspect fibrosis of underlying tissue due to subcutaneous  indentation. Ulcer 2x3 cm^2^. No signs of regrowth. | Treatment of ulcer: honey ointment.  Euthanized 21.5 months after treatment due to unrelated disease. No signs of recurrence at this point. |
| **16** | Histiocytic sarcoma right elbow, microscopic disease. Treated 1.5 months post-surgery. | Mild erythema over the scar. No signs of regrowth. | No sign of regrowth. Alopecia and patchy pigmentation of skin in RT field. | Alopecia and hyperpigmentation. Dermis superficially slightly thickened locally in distal part of scar. No sign of tumor regrowth | Pigmented and slightly thickened skin on elbow laterally as before, very mild erythema. No signs of tumor regrowth. | X-ray and ultrasound with no signs of metastasis. X-rays of treatment site: no changes. No signs of tumor regrowth. | Adjunctive treatment with CCNU (Lomustine) after radiation therapy. Alive at 1093 days after treatment. |

RT = radiotherapy, MCT = Mast cell tumor, STS = Soft tissue sarcoma, SCC = squamous cell carcinoma, CR = complete response, PR = partial response, SD = stable disease, PD = progressive disease, FNA = fine needle aspiration, CT = Computed Tomography, NSAID = Non-Steroidal Anti-Inflammatory Drug

**Table 2:** VRTOG v2/v1 adverse effects

|  | Type | Dose (Gy) | Field size (cm) | Day 7 | 1 mo | 3 mo | 6 mo | 12 mo | Worst AE | Bone in RT field | ORN | Related comments |
| --- | --- | --- | --- | --- | --- | --- | --- | --- | --- | --- | --- | --- |
| 1 | STS | 15 | 8x4 | 0/0 | 1/1 | 1/1 | 0/0 | 0/0 | 1/1 | Yes | No |  |
| 2 | STS | 16 | 2x6 | 0/0 | 1/1 | 0/0 | - | - | 1/1 | Yes | No | A small ulcer present at 1 month thought to be suture reaction |
| 3 | Plasmacytoma | 20 | 2 Ø | 0/0 | 0/0 | 0/0 | 1/1 | 0/0 | 1/1 | Yes | No |  |
| 4 | MCT | 20 | 4x6 | 1/1 | 1/1 | 1/1 | - | 1/1 | 1/1 | Yes | No |  |
| 5 | STS | 25  35 | 5 Ø | 0/0 | 0/0 | 1/1 | 1/1 | 1/1 | 1/1 | Yes | No |  |
| 6 | MCT | 30 | 2 Ø | 0/0 | 1/1 | 1/1 | 1/1 | 1/1 | 1/1 | Yes | No |  |
| 7 | MCT | 30 | 2 Ø | 0/0 | 1/1 | 1/1 | 1/1 | 1/1 | 1/1 | No | - |  |
| 8 | MCT | 30 | 3 Ø | 0/0 | 1/1 | 1/1 | 1/1 | - | 1/1 | Yes | No | Lost claw capsule on digit II before 3-month checkup |
| 9 | STS | 30 | 5 Ø | 0/0 | 1/1 | - | 1/1 | - | 1/1 | Yes | No |  |
| 10 | STS | 30 30 | 10x10  8 Ø | 1/1 | 1/1 | - | - | - | 1/1 | No | No |  |
| 11 | SCC | 35 | 2x5 | 0/0 | 4/3 | 4/3 | 1/1 | - | 4/3 | Yes | No | Ulceration did not heal until 6mo due to autotrauma (licking) |
| 12 | MCT | 35 | 5 Ø | 0/0 | 1/1 | 1/1 | - | - | 1/1 | No | - |  |
| 13 | MCT | 35 | 6 Ø | 0/0 | 2/2 | 4/3 | - | - | 4/3 | Yes | No | Ulceration due to autotrauma (licking) |
| 14 | SCC | 35 | 6 Ø | 0/0 | 4/3 | 4/3 | - | - | 4/3 | Yes | No | Ulcer present at pretreatment, foot pad detached after 1 month |
| 15 | MCT | 35 | 8x4 | 4/3 | 4/3 | 3/3 | 1/1 | 4/3 | 4/3 | Yes | No | Ulcer present at pretreatment, worsens after treatment |
| 16 | Histiocytic sarcoma | 35 | 10x4 | 1/1 | 1/1 | 1/1 | 1/1 | 1/1 | 1/1 | Yes | No |  |

X / Y where X = VRTOG v2 and Y = VRTOG v1. ORN = osteoradionecrosis, mo = month(s)
MCT = Mast cell tumor, STS = soft tissue sarcoma, SCC = squamous cell carcinoma
Acute toxicity = 0-3 months. Chronic toxicity = 3 months forward.

**Table 3:** RECIST scoring and survival time

|  | Type and treatment setting | Dose (Gy) | Day 7 | 1 mo | 3 mo | 6 mo | 12 mo | Best response | PD | Survival time (days) | PFS (days) | Related comments |
| --- | --- | --- | --- | --- | --- | --- | --- | --- | --- | --- | --- | --- |
| 1 | STS (micro) | 15 | NR | NR | NR | NR | NR | NR | No | 1020^a^ | 1020^a^ |  |
| 2 | STS (micro) | 16 | NR | NR | NR | - | - | NR | No | 932^a^ | 932^a^ |  |
| 3 | Plasmacytoma (macro) | 20 | SD | SD | SD | PR | CR | CR | No | 677 | 677 |  |
| 4 | MCT (micro) | 20 | NR | NR | NR | - | NR | NR | No | 659 | 659 |  |
| 5 | STS (macro) | 25  35 | SD | SD | SD | SD | PR | PR | No | 677 | 677 |  |
| 6 | MCT (macro) | 30 | PR | CR | CR | CR | CR | CR | No | 1266^a^ | 1266^a^ | Microscopic evidence of residual disease |
| 7 | MCT (macro) | 30 | PR | CR | CR | CR | CR | CR | No | 1266^a^ | 1266^a^ |  |
| 8 | MCT (micro) | 30 | NR | NR | NR | NR | - | NR | No | 321 | 321 |  |
| 9 | STS (micro) | 30 | NR | NR | - | NR | - | NR | No | 1196^a^ | 1196^a^ |  |
| 10 | STS (macro) | 30 | SD | PR | - | - | - | PR | No | 63 | 63 | No local progression, but death due to distant metastasis |
| 11 | SCC (macro) | 35 | SD | CR | CR | CR | - | CR | Yes | 308 | 296 | Euthanized due to PD inside RT field |
| 12 | MCT (macro) | 35 | PR | PD | PD | - | - | PR | Yes | 152 | 36 | PD inside of RT field, mastinib added at PD. Euthanized due to PD |
| 13 | MCT (macro) | 35 | SD | PR | CR | - | - | CR | Yes | 136 | 116 | Euthanized due to PD outside RT field |
| 14 | SCC (macro) | 35 | SD | PR | - | - | - | PR | Yes | 88 | 77 | Euthanized due to PD inside RT field |
| 15 | MCT (micro) | 35 | NR | NR | NR | NR | NR | NR | No | 650 | 650 |  |
| 16 | Histiocytic sarcoma (micro) | 35 | NR | NR | NR | NR | NR | NR | No | 1093^a^ | 1093^a^ | Additional therapy: CCNU + corticosteroids |

a) Patient alive at the time of writing.
PD = progressive disease, SD = stable disease, PR = partial response, CR = complete response, PFS = progression-free survival, mo = month(s), NR = no recurrence, MCT = mast cell tumor, STS = soft tissue sarcoma, SCC = squamous cell carcinoma
